# Detailed Analysis of the African Green Monkey Model of Nipah Virus Disease

**DOI:** 10.1371/journal.pone.0117817

**Published:** 2015-02-23

**Authors:** Sara C. Johnston, Thomas Briese, Todd M. Bell, William D. Pratt, Joshua D. Shamblin, Heather L. Esham, Ginger C. Donnelly, Joshua C. Johnson, Lisa E. Hensley, W. Ian Lipkin, Anna N. Honko

**Affiliations:** 1 Virology Division, United States Army Medical Research Institute of Infectious Diseases, 1425 Porter Street, Fort Detrick, Maryland, United States of America; 2 Center for Infection and Immunity, Columbia University Mailman School of Public Health, 722 W. 168^th^ Street, New York, New York, United States of America; 3 Pathology Division, United States Army Medical Research Institute of Infectious Diseases, 1425 Porter Street, Fort Detrick, Maryland, United States of America; Virginia Polytechnic Institute and State University, UNITED STATES

## Abstract

Henipaviruses are implicated in severe and frequently fatal pneumonia and encephalitis in humans. There are no approved vaccines or treatments available for human use, and testing of candidates requires the use of well-characterized animal models that mimic human disease. We performed a comprehensive and statistically-powered evaluation of the African green monkey model to define parameters critical to disease progression and the extent to which they correlate with human disease. African green monkeys were inoculated by the intratracheal route with 2.5×10^4^ plaque forming units of the Malaysia strain of Nipah virus. Physiological data captured using telemetry implants and assessed in conjunction with clinical pathology were consistent with shock, and histopathology confirmed widespread tissue involvement associated with systemic vasculitis in animals that succumbed to acute disease. In addition, relapse encephalitis was identified in 100% of animals that survived beyond the acute disease phase. Our data suggest that disease progression in the African green monkey is comparable to the variable outcome of Nipah virus infection in humans.

## Introduction

Nipah virus (NiV) is a recently emerged member of the family *Paramyxoviridae* that, together with Hendra virus (HeV), comprises the genus *Henipavirus*. The henipaviruses are zoonotic viruses that infect and cause fatal disease in a wide variety of animal species including humans, swine, horses, dogs, and cats [1]. The natural reservoir of henipaviruses is the fruit bat of the genus *Pteropus;* however, neutralizing antibodies to NiV have been detected in other non-*Pteropus* fruit bat species from Cambodia, Thailand, Indonesia, Bangladesh, Madagascar, and West Africa [2,3]. Due to slash and burn agriculture in Asia and Australia, *Pteropus* bats are coming into increasing proximity to humans and livestock; zoonotic transmission may occur by ingestion of fruits and saps that have been contaminated by bat excreta, or through contact with animals infected with NiV [4–8].

Henipaviruses are classified as priority pathogens in Category C by the Centers for Disease Control and Prevention and as select agents by the Division of Select Agents and Toxins. NiV was first identified in Malaysia and Singapore in 1998, and resulted in approximately 300 human cases of febrile encephalitis with pneumonitis and a mortality rate of 40% [9,10]. Since 1998, outbreaks of NiV have been reported in Bangladesh and India with mortality rates approaching 78% [11,12]. In addition to zoonotic transmission, the number of human-to-human transmission events noted for NiV has increased, with evidence suggesting that the virus is shed in saliva, nasopharyngeal secretions, and urine [7,13–16].

NiV infection in humans presents as an acute encephalitis accompanied by fever, headache, drowsiness, dizziness, myalgia, and vomiting [17]. In a large scale study reviewing autopsy results from 32 individuals infected with NiV (reviewed in [18]), systemic vasculitis was observed and vasculitis-induced thrombosis and parenchymal necrosis was prominent in the brain. Additional findings included syncytial multinucleated endothelial cells, the presence of NiV antigen in cerebral vascular endothelium, and direct NiV infection of neurons in the brain. Lymphocytolysis and lymphoid depletion in the lymph nodes, spleen, and thymus have also been reported [19]. In fatal cases, death occurs between 1–2 weeks following initial symptom onset [20], and 20% of survivors have residual neurologic effects. Relapse/late onset encephalitis occurs in approximately 7.5% of survivors and 3.4% of individuals who were initially asymptomatic, and can occur from several months to up to 4 years following initial exposure [21]. Neuronal injury is extensive and viral inclusions are prominent, suggesting that relapse/late onset encephalitis is the result of reactivation of the previous neuronal NiV infection [18]. The mortality rate of relapse/late onset encephalitis is approximately 18%; 61% of survivors have neurologic sequellae [21].

As there are no approved active or passive therapeutic modalities for henipavirus infection, the development of effective therapeutics to treat NiV infection is critical to protect against cases resulting from a natural outbreak, laboratory mishap, or deliberate misuse. Paramount to this effort is the development and characterization of models that accurately depict human disease. To this end, we performed a characterization study in African green monkeys using the Malaysia strain of NiV. African green monkeys were chosen because this was the only nonhuman primate model of nipah that had been described in the literature, and the animals developed clinical disease similar to what has been described for humans [22]. Animals inoculated by the intratracheal (i.t.) route with 2.5×10^4^ plaque forming units (pfu) of NiV that succumbed to disease developed severe respiratory distress and shock. Animals that survived to the end of study had chronic vasculitis and encephalitis, with viral antigen staining in the brain. The findings were, therefore, consistent with those reported for human NiV disease.

## Results

### Lethality of NiV-Malaysia in African Green Monkeys

**Fig 1:**
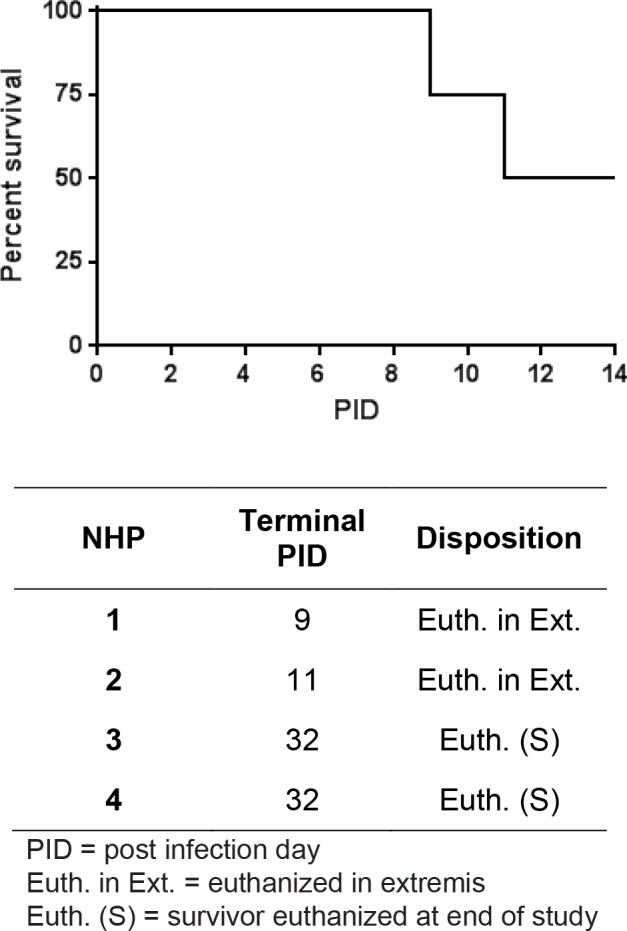
Percent survival. Four animals were inoculated with 2.5×10^4^ pfu of the Malaysia strain of NiV by the i.t. route. The study percent survival, the day of disposition, and the method of disposition for animals on study are shown. PID = post infection day.

Four healthy, henipavirus-naïve African green monkeys were inoculated by the i.t. route with 2.5×10^4^ pfu of NiV (Malaysia strain). Animals were observed at least twice daily for clinical signs of illness and were humanely euthanized when moribund; two of the animals became moribund and were euthanized on PID 9 (nonhuman primate [NHP] 1) and 11 (NHP 2) (Fig. 1). The remaining two animals (NHP 3 and 4) survived until the end of study (PID 32) at which time they were humanely euthanized; all surviving animals were free of disease signs at the time of euthanasia.

### Clinical Observations

Clinical observations are summarized in Table 1. Cage side observations of un-anesthetized animals were performed at least twice daily, and physical examinations on anesthetized animals were performed on all blood collection days (PID-8, -1, 0, 1, 3, 5, 7, 10, 12, 14, and day of disposition). In addition, animals were implanted with ITS telemetry implants for hourly measurement of heart rate, respiratory rate, blood pressure, and core body temperature. Transient core body temperature reductions during the time of blood collections were graphed but excluded from further analysis as drops in body temperature can occur under anesthesia.

**Table 1:** Clinical Observations.

	NHP 1	NHP 2	NHP 3	NHP 4
Decreased Responsiveness (AO)	+	+	-	+
Tachypnea (AO)	+	+	-	+
Tremors (AO)	-	+	-	-
Exudate (AO)	+	-	-	+
Lymphadenopathy (PE)	+	+	+	+
Weight Loss (PE)	-	-	-	+
Fever (T)	+	+	+	+
Hypothermia (T)	+	+	-	-
Tachycardia (T)	+	+	+	+
Tachypnea (T)	+	+	NA	+
Hypotension (T)	+	+	-	+

AO = awake (un-anesthetized) observation finding.

PE = physical examination finding.

T = telemetry finding.

NA = not assessed.

**Fig 2:**
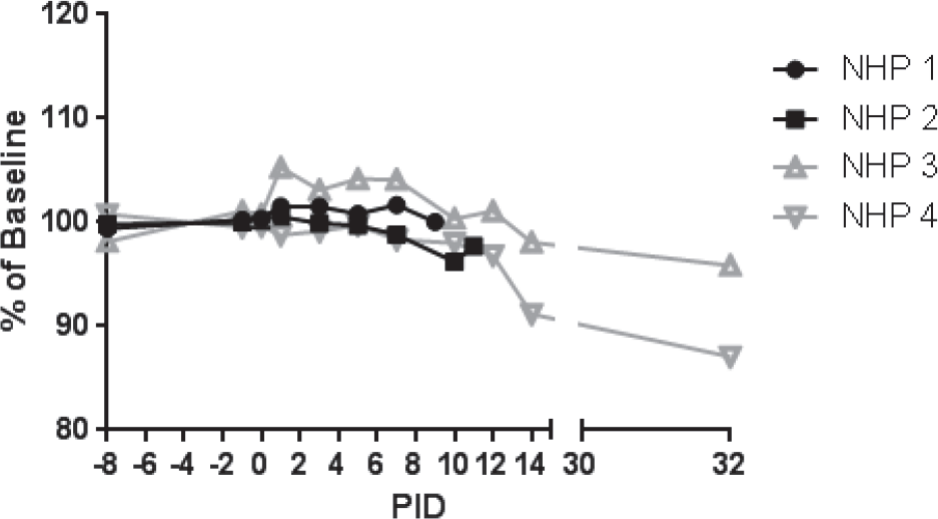
Weight change for NiV-infected animals. Weights for animals on study are shown as a percent of baseline (mean of the PID-8, -1, and 0 values for that animal). Black lines and symbols represent animals that succumbed, and grey lines and symbols represent animals that survived to end of study.

**Fig 3:**
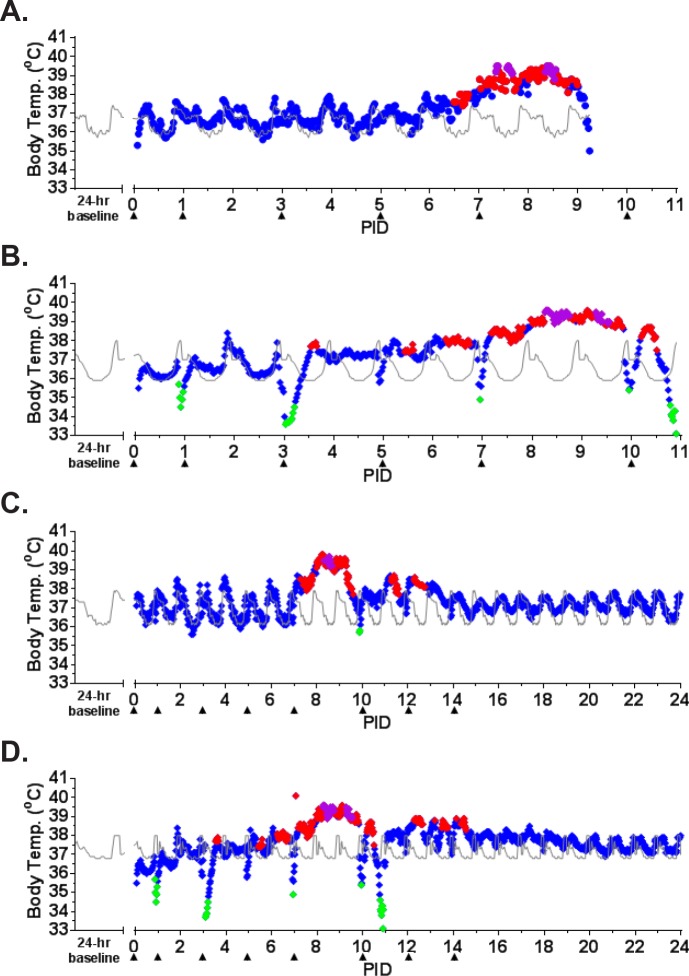
Core body temperature as measured by telemetry. ITS telemetry devices were used to collect core body temperature data. (A) NHP 1; (B) NHP 2; (C) NHP 3; (D) NHP 4. The grey line represents baseline (mean of the telemetry data collected in the six days prior to challenge) for each animal. The diamonds represent fever (♦; > 1.5°C above baseline), hyperpyrexia (♦; > 3°C above baseline), and hypothermia (♦; > 2°C below baseline), and normal body temperature values (♦; a temperature falling between fever and hypothermia).

**Fig 4:**
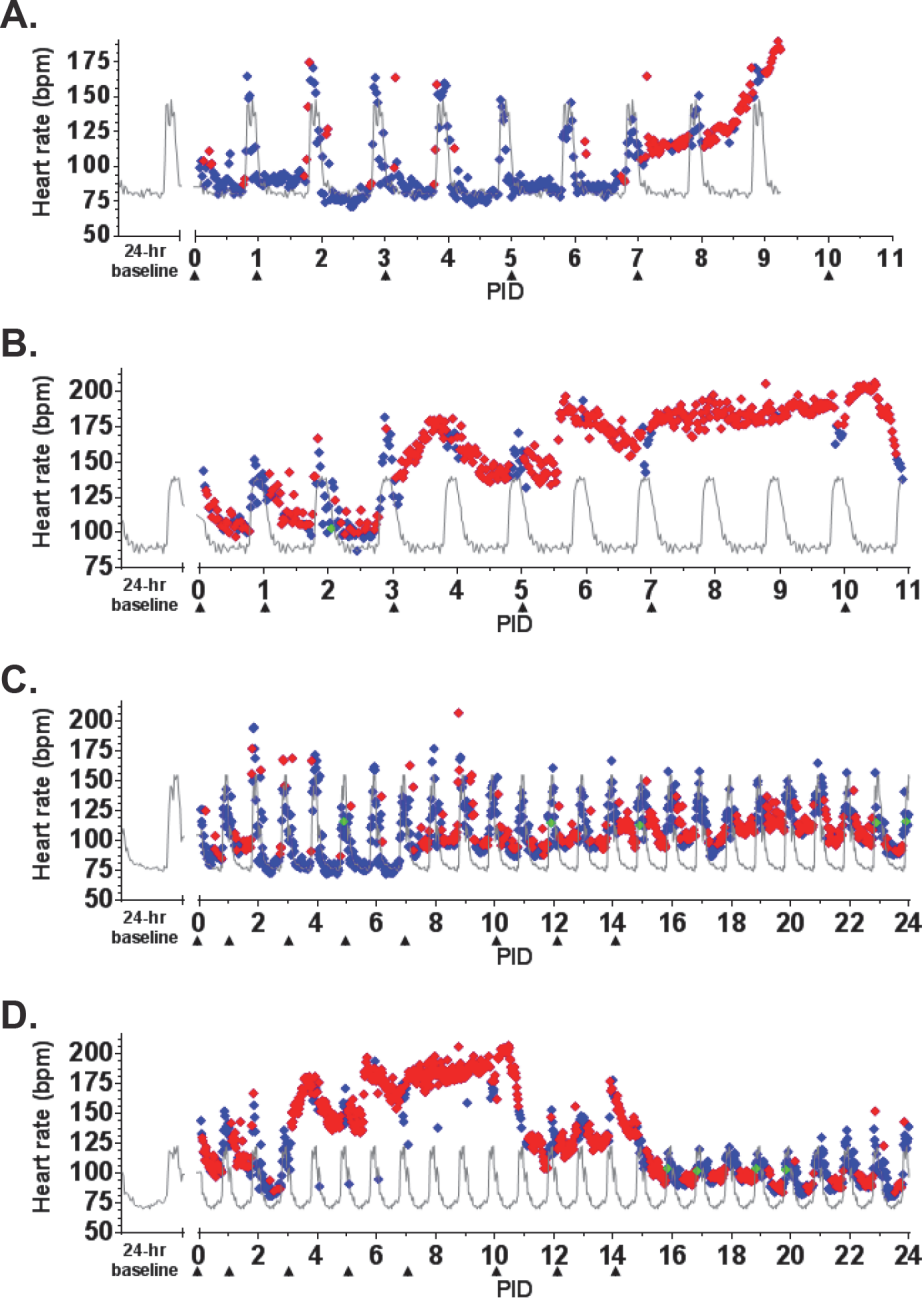
Heart rate as measured by telemetry. ITS telemetry devices were used to collect heart rate data. (A) NHP 1; (B) NHP 2; (C) NHP 3; (D) NHP 4. The grey line represents baseline (mean of the telemetry data collected in the six days prior to challenge) for each animal. The diamonds represent statistically significant heart rate values (> +3 SD [♦] above or > -3 SD [♦] below baseline) or non-significant values (♦). bpm = beats per minute.

**Fig 5:**
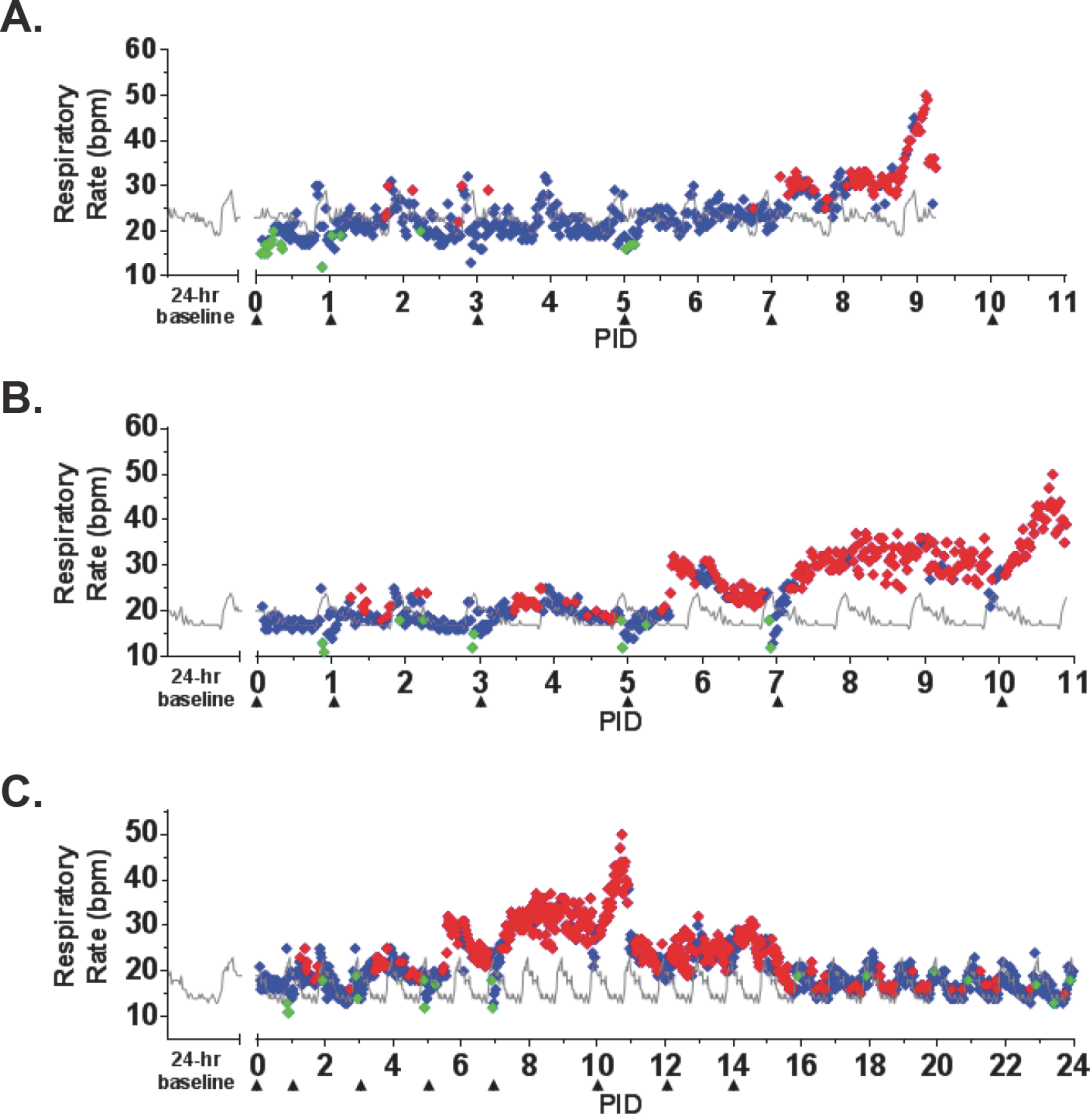
Respiratory rate as measured by telemetry. ITS telemetry devices were used to collect respiratory rate data. (A) NHP 1; (B) NHP 2; (C) NHP 4. The grey line represents baseline (mean of the telemetry data collected in the six days prior to challenge) for each animal. The diamonds represent statistically significant respiratory rate values (> +3 SD [♦] above or > -3 SD [♦] below baseline) or non-significant values (♦). bpm = beats per minute.

**Fig 6:**
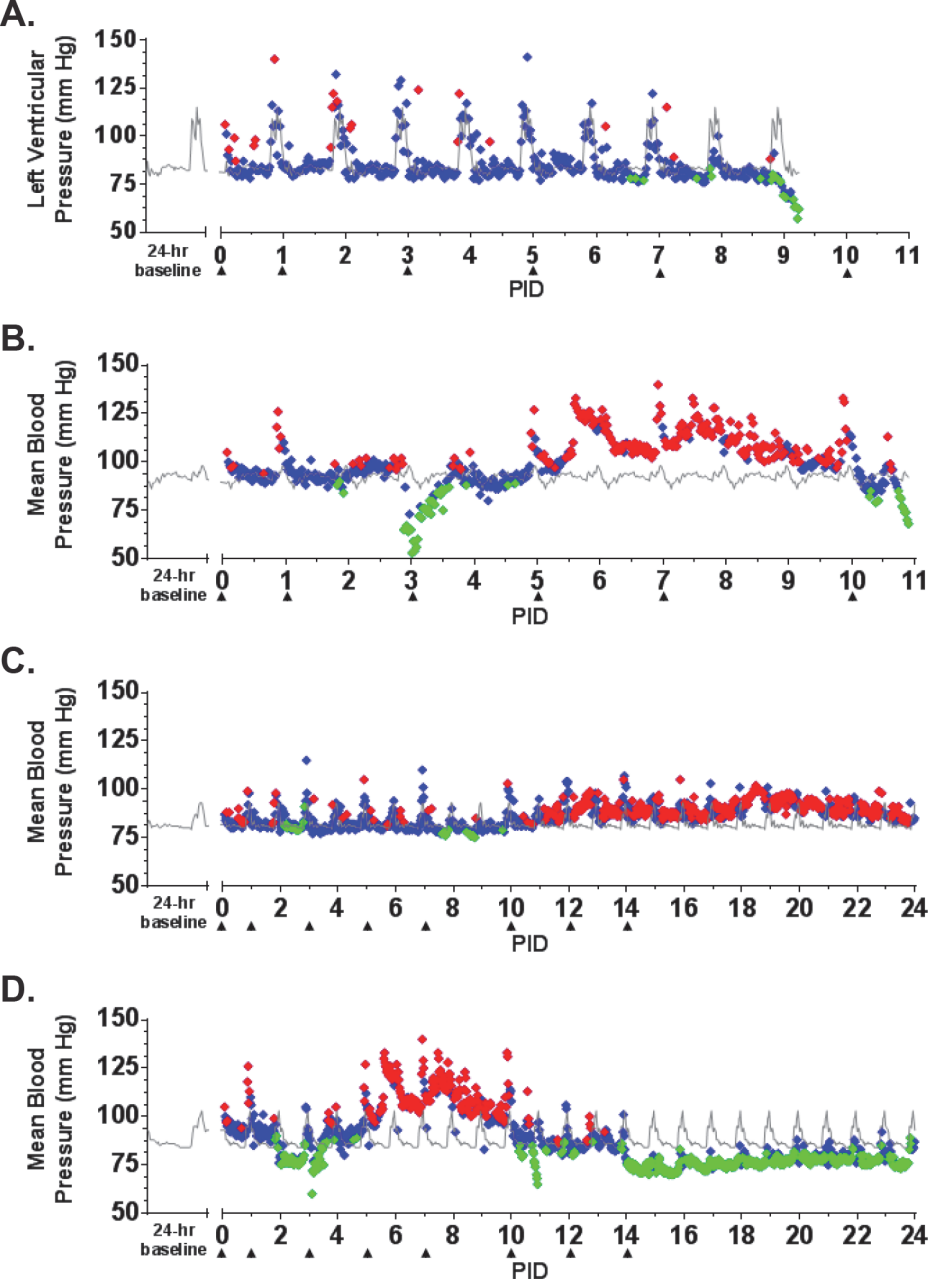
Blood pressure as measured by telemetry. ITS telemetry devices were used to collect blood pressure data. (A) NHP 1; (B) NHP 2; (C) NHP 3; (D) NHP 4. The grey line represents baseline (mean of the telemetry data collected in the six days prior to challenge) for each animal. The diamonds represent statistically significant blood pressure values (> +3 SD [♦] above or > -3 SD [♦] below baseline) or non-significant values (♦).

The earliest sign of disease was moderate to severe lymphadenopathy of the axillary lymph nodes, typically appearing at PID 5; axillary lymph nodes were > 20 mm in size (normal average size is approximately 6 mm) at the time of disposition for animals that succumbed. By PID 9, animals were unresponsive and tachypneic. NHP 1 had bloody exudate in its nose and mouth at the time of disposition (PID 9). NHP 2 developed tremors on PID 10. Neither of these animals had significant weight loss (Fig. 2). Core body temperature (as determined by telemetry) was elevated by PID 6–7 for NHP 1 and 2, with a sharp drop in temperature observed at the time that the animals became moribund (Fig. 3A-B). Telemetry also revealed an increase in heart rate (PID 7–9 for NHP 1 and PID 3–11 for NHP 2) and a corresponding increase in respiratory rate (PID 7–9 for NHP 1 and PID 6–11 for NHP 2) (Figs. 4A-B, 5A-B). Blood pressure data was more variable (Fig. 6A-B). For NHP 1, blood pressure fell below baseline (mean of data collected during the six days prior to challenge) as the animal became moribund (PID 9). Fluctuations in blood pressure were observed for NHP 2. A drop below baseline was noted for this animal on PID 3, but pressure had returned to normal by PID 4. Based on the transient nature of the change, this drop in blood pressure on PID 3, which occurred on a phlebotomy day, may be attributable to an anesthesia effect rather than NiV disease. A subsequent elevation in blood pressure was observed between PID 6–10; similar to NHP 1, blood pressure fell below baseline as the animal became moribund (PID 11).

NHP 3 and 4 survived to end of study. No overt signs of disease were apparent in NHP 3 through cage side observation; however, moderate axillary lymphadenopathy was noted on PID 5. Telemetry also revealed an increase in core body temperature of similar magnitude to the other three animals between PID 7–9 (Fig. 3C) as well as modest elevations in heart rate (PID 7–24; Fig. 4C) and blood pressure (PID 11–24; Fig. 6C); respiratory rate was not recorded for this animal due to an issue with the intrathoracic pressure sensor.

NHP 4 developed clinical illness similar to both animals that succumbed. This animal had severe axillary lymphadenopathy by PID 5; moderate axillary lymphadenopathy was noted as late as PID 12. This animal became less responsive and had labored breathing on PID 9–10; nasal exudate was noted on PID 10. NHP 4 was the only animal that lost weight compared to baseline, losing 0.5 kg by PID 14 and 0.7 kg by PID 32 (Fig. 2). It also had an increase in core body temperature similar to animals that succumbed, and this was noted on PID 6–10 (Fig. 3D). Heart rate was elevated on PID 3–15 (highest rates were observed on PID 3–10) and corresponded with an increased respiratory rate during that same timeframe (Figs. 4D, 5D). Similar to NHP 2, which succumbed, a drop in blood pressure below baseline on PID 2–3 was followed by an elevation in blood pressure on PID 5–10 (Fig. 6D). Blood pressure returned to baseline between PID 11–14, and then fell below baseline and remained below baseline until the end of telemetry data collection (PID 24) (Fig. 6D).

## Viral Load and Neutralizing Antibody Analyses

**Fig 7:**
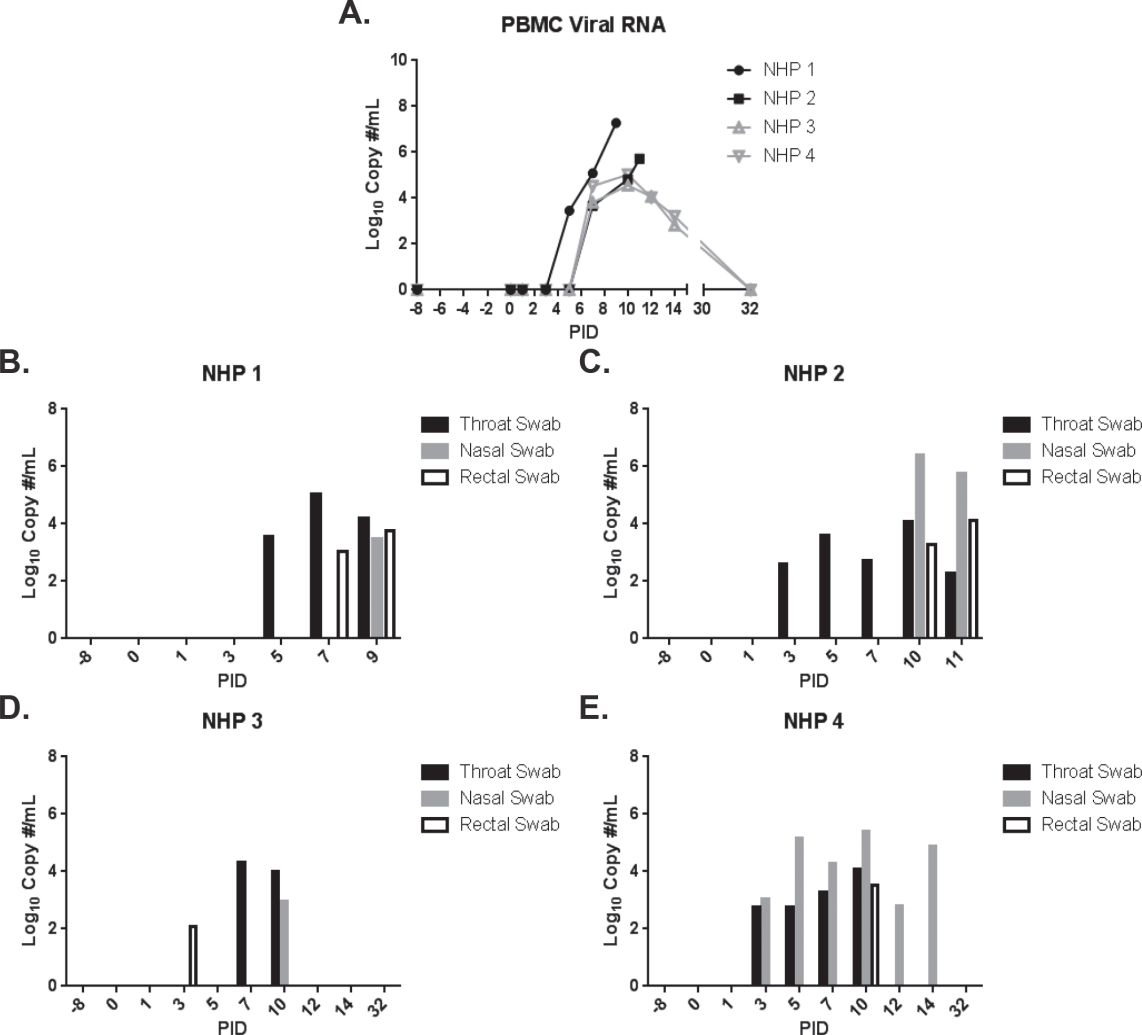
Viral RNA in PBMC and throat, nasal, and rectal swabs as measured by qRT-PCR. NiV-specific qRT-PCR was performed on RNA extracted from PBMC samples and throat, nasal, and rectal swab clarified homogenates. The results for PBMC samples are shown in (A); black lines and symbols represent animals that succumbed, and grey lines and symbols represent animals that survived to end of study. The results for throat (black bars), nasal (grey bars), and rectal (white bars with black outline) swabs are shown in (B)-(E).

Quantitative reverse transcription polymerase chain reaction (qRT-PCR) was performed on peripheral blood mononuclear cells (PBMC) isolated from EDTA whole blood (Fig. 7A). Viral RNA was first detected on PID 5 for NHP 1 (succumbed on PID 9). All animals had PBMC-associated NiV RNA by PID 7, confirming that all animals were infected. Peak levels of 5.7–7.3 Log_10_ copies/mL were measured on the day of disposition for animals that succumbed, compared to 4.5–5.0 Log_10_ copies/mL measured on PID 10 for animals that survived. Levels steadily declined to approximately 3.0 Log_10_ copies/mL on PID 14, 7 days following the first detection of viral RNA in the surviving animals; PBMC-associated NiV RNA was not detected on PID 32.

The presence of viral RNA in throat, nasal, and rectal swabs was also assessed by qRT-PCR (Fig. 7B-E). Viral RNA was detected for all three swab types on at least 1 day for all animals on this study; peak levels were not dramatically different between survivors and non-survivors. With only a few exceptions, viral RNA was detected earliest in throat swabs (around PID 3–5), and later (around PID 7–10) in nasal and rectal swabs. For surviving animals, swab-associated viral RNA was generally not detected beyond PID 10.

To assess the viability of virus in swab samples, plaque assays were performed, and the number of pfu/mL of clarified culture was determined (Table 2). Infectious virus was not detected in swab samples from animals that survived to end of study. Animals that succumbed had measureable infectious virus in throat swabs; virus was detected earliest on PID 5 for NHP 2, and was present for both animals by PID 7. Additionally, infectious virus was detected in nasal swabs for NHP 2 (PID 10–11) and a rectal swab for NHP 1 (PID 9).

**Table 2:** Infectious NiV in Throat, Nasal, and Rectal Swab Samples.

	PID	pfu/mL
*Throat Swab Samples*		
NHP 1	7	5.0×10^1^
NHP 1	9	7.5×10^1^
NHP 2	5	5.0×10^1^
NHP 2	7	7.5×10^1^
NHP 2	10	2.5×10^1^
NHP 2	11	2.5×10^1^
*Nasal Swab Samples*		
NHP 2	10	1.3×10^2^
NHP 2	11	7.5×10^1^
*Rectal Swab Samples*		
NHP 1	9			2.0×10^2^

PID = post infection day.

**Fig 8:**
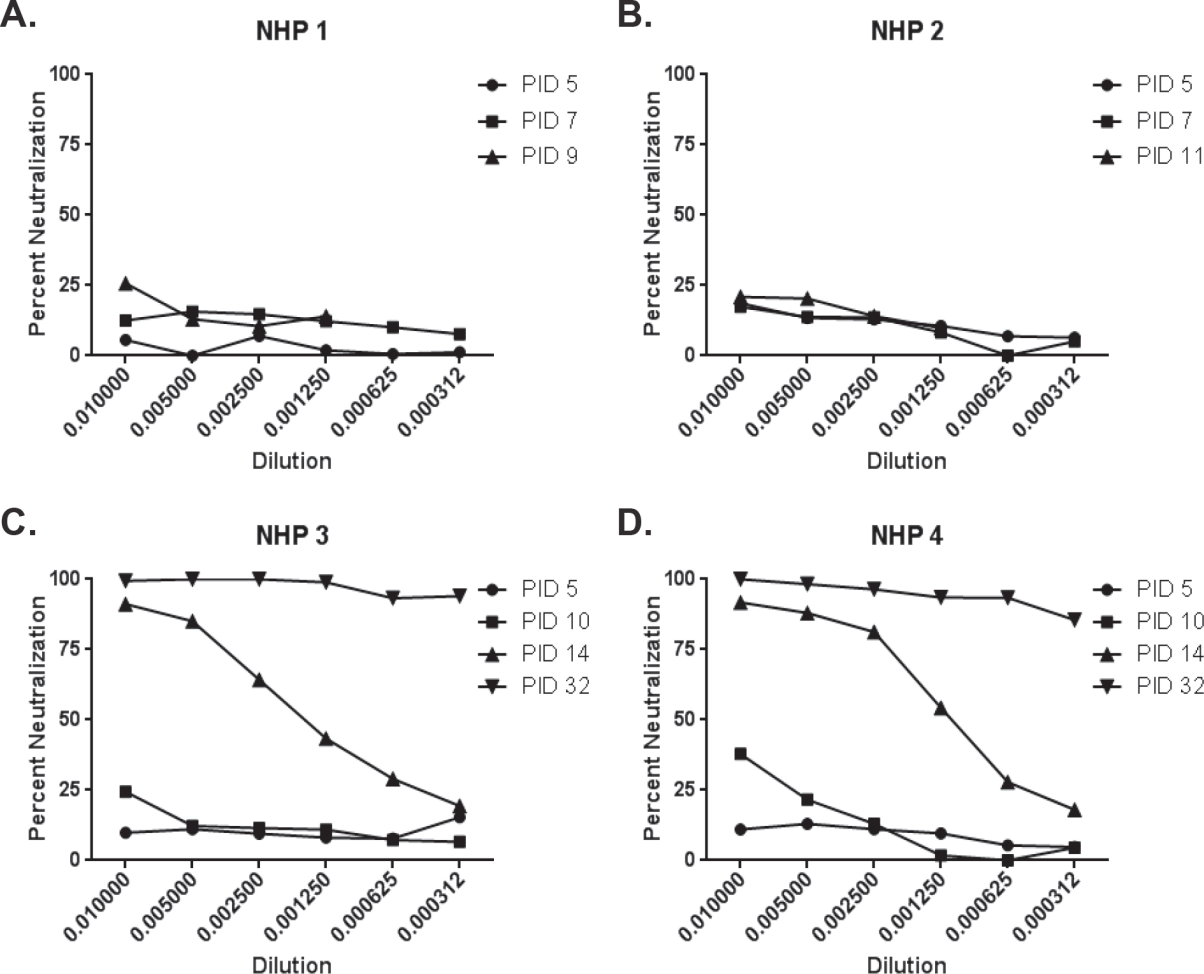
Percent neutralization of pseudotype virus. A VSV-pseudotype neutralization assay was performed using serum, and percent neutralization is shown. The PID that serum was analyzed is shown in the figure legend. (A) NHP 1; (B) NHP 2; (C) NHP 3; (D) NHP 4.

To assess the neutralizing antibody response in African green monkeys infected with NiV, we performed a VSV-pseudotype neutralization assay (Fig. 8). Surviving animals (NHP 3 and 4) developed a robust neutralizing antibody response between PID 10–14, with ≥ 90% neutralization observed at the 1:3200 dilution by PID 32 (Fig. 8C-D. Animals that succumbed (NHP 1 and 2) were not able to mount a strong neutralizing antibody response prior to becoming moribund, with less than 30% neutralization observed at the lowest dilution tested (1:100) (Fig. 8A-B).

### Clinical Pathology

**Fig 9:**
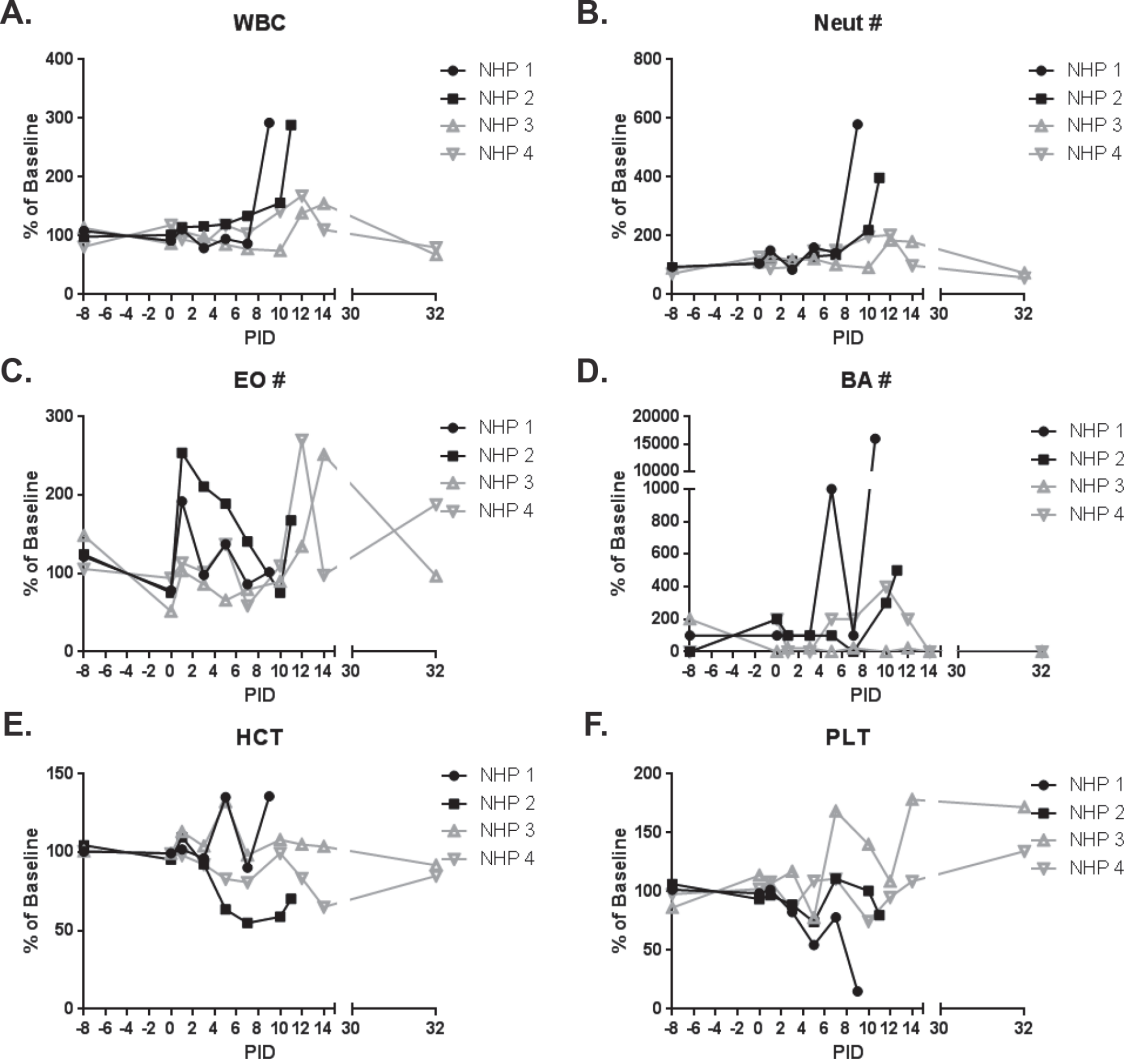
Hematology. Hematology was performed on EDTA whole blood using a Hemavet instrument. Hematology measurements for animals on study are shown as a percent of baseline (mean of the PID-8, -1, and 0 values for that animal). Black lines and symbols represent animals that succumbed, and grey lines and symbols represent animals that survived to end of study. (A) WBC = white blood cells; (B) Neut = neutrophils; (C) EO = eosinophils; (D) BA = basophils; (E) HCT = hematocrit; (F) PLT = platelets.

**Fig 10:**
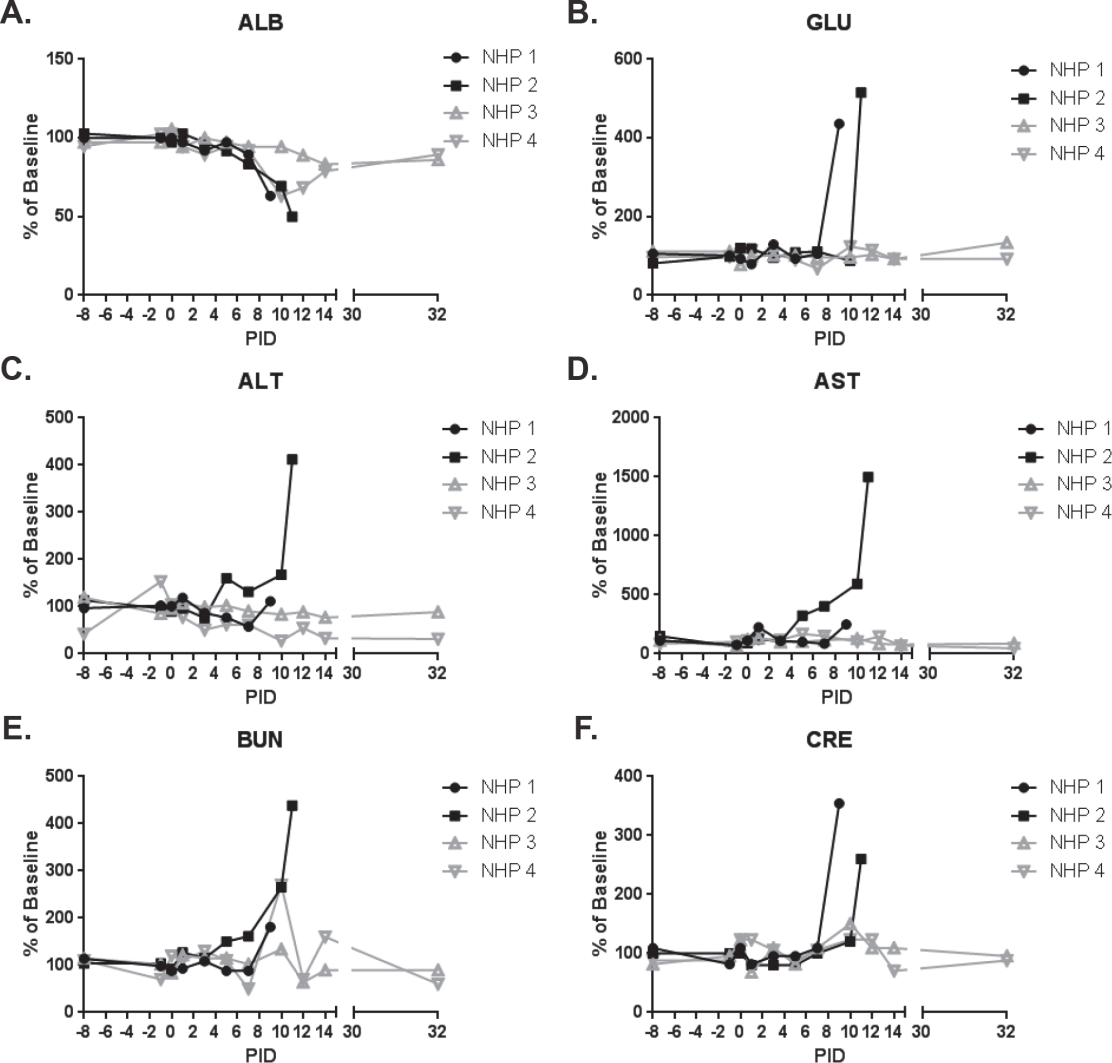
Clinical chemistries. Clinical chemistries were performed on serum using a Piccolo Point-Of-Care instrument. Clinical chemistry measurements for animals on study are shown as a percent of baseline (mean of the PID-8, -1, and 0 values for that animal). Black lines and symbols represent animals that succumbed, and grey lines and symbols represent animals that survived to end of study. (A) ALB = albumin; (B) GLU = glucose; (C) ALT = alanine transaminase; (D) AST = aspartate transaminase; (E) BUN = blood urea nitrogen; (F) CRE = creatinine.

Hematology (Fig. 9) and clinical chemistries (Fig. 10) were performed on whole blood and serum, respectively. Leukocytosis associated with a neutrophilic and basophilic granulocytosis was noted for animals that succumbed to infection; peak levels were measured on the day of disposition. Slight elevations in white blood cells and neutrophils were also observed for survivors on PID 10–14, and basophilia was observed for NHP 4 on PID 10–14; levels returned to baseline by PID 32. Additionally, eosinophilia was observed for survivors on PID 12–14.

Hematocrit was reduced for NHP 2 (PID 5–11), which succumbed. Platelets were reduced by PID 5 for both animals that succumbed. For NHP 1, a slight rebound in platelets on PID 7 was followed by a huge drop on PID 9. Similarly, a rebound in platelets was observed for NHP 2 on PID 7–10 with another drop observed on PID 11. Albumin was substantially reduced on the day of disposition for both animals that succumbed which, together with reduced hematocrit and platelet levels, suggested coagulopathy and hemorrhage. Reductions in hematocrit (PID 14), albumin (PID 10–14), and platelets (PID 10) were also seen for NHP 4 (end of study survivor); levels had returned to near baseline by PID 32. Platelets were elevated for NHP 3 by PID 7 and, aside from a drop to near baseline on PID 12, remained high through the end of study.

Elevations in alanine transaminase and aspartate transaminase for NHP 2 at the time of disposition (PID 11) were suggestive of hepatocyte damage; changes in alanine transaminase and aspartate transaminase were not noted for NHP 1 and both survivors. Glucose levels were elevated on the day of disposition for animals that succumbed; similar elevations were not noted for end of study survivors.

Azotemia is characterized by elevated levels of nitrogen-containing compounds in the blood, and is differentially diagnosed by measuring the levels of blood urea nitrogen (BUN) and creatinine (CRE). Both animals that succumbed had elevated levels of BUN and CRE on the day of disposition consistent with azotemia. NHP 4 had an elevation in BUN (levels of CRE remained near baseline) on PID 10, which was most likely indicative of mild hypovolemia not associated with azotemia.

### Histopathology and Immunohistochemistry

**Fig 11:**
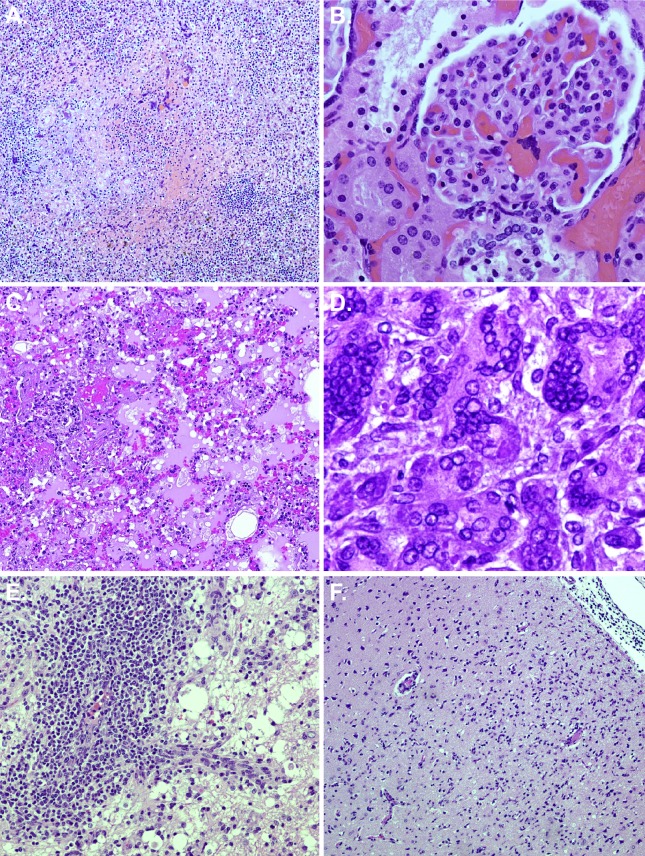
Histopathology. Necropsies and histopathology were performed on all animals. (A) NHP 1: Spleen—10x magnification, splenitis with hemorrhage, necrosis, and numerous syncytial cells; (B) NHP 1: Kidney (glomerulus) – 40x magnification, syncytial cells; (C) NHP 2: Lung—10x magnification, interstitial pneumonia; (D) NHP 2: Pancreas—60x magnification, multiple viral syncytial cells with intranuclear and intracytoplasmic inclusions; (E) NHP 3: Cerebellum—20x magnification, lymphoplasmacytic and histocytic vasculitis and encephalitis with numerous gitter cells; (F) NHP 4: Cerebrum—10x magnification, encephalitis and meningitis.

At necropsy, animals that succumbed had red, mottled, and edematous lungs (all lobes equally affected), mediastinal edema, pleural effusion (approximately 10 mL of red-tinged serous fluid was present), and hepatic pallor, mottling, and/or enlargement. Necropsies were performed to collect a complete set of tissue samples for histology and immunohistochemistry (IHC). Representative histopathological images are shown in Fig. 11.

Lesions were present in most tissues examined, and these lesions were generally centered on small vessels. Affected vessels often had transmural vasculitis with an accumulation of mixed populations of mononuclear cells within the tunica adventitia. These small caliber vessels were often lined by endothelial syncytia. The adjacent tissue was often filled with hemorrhage, fibrin, and edema. Some vessels also developed thrombi which led to adjacent parenchymal necrosis.

The most prominent lesions in the animals included: interstitial lymphohistiocytic pneumonia with hemorrhage; systemic lymphohistiocytic vasculitis; necrohemorrhagic splenitis of the white pulp; gliosis with rarefaction and spongiosis +/− necrosis; necrohemorrhagic pancreatitis; necrohemorrhagic lymphadenitis +/− sinus histiocytosis of the mediastinal, tracheobronchial, and inguinal lymph nodes; sinus histiocytosis +/− lymphoid depletion of the axillary, mesenteric, and submandibular lymph nodes; glomerular fibrosis and lymphohistiocytic inflammation in the kidney; necrohemorrhagic thymitis; necrohemorrhagic tonsillitis. In addition, NHP 1 had granulomatous myositis of the skeletal muscle, and NHP 2 had necrosis in the liver and thyroid gland. Syncytial cells, which are a common finding following NiV infection [23], were present in numerous tissues including the lung, spleen, kidney, pancreas, skeletal muscle, mesenteric lymph node, and axillary lymph node. Fibrin deposition was present in the following: lungs; mediastinal, tracheobronchial and axillary lymph nodes; spleen; pancreas; thymus; adrenal gland; tonsils; and liver. Mild or minimal lymphohistiocytic or lymphoplasmacytic inflammation was noted in the adrenal gland, tongue, submandibular salivary gland, larynx, and liver. Most tissues examined were positive for NiV antigen by IHC (Table 3), with the strongest staining (> 40 cells/high power field [hpf]) observed in the tonsil, lung, thymus, mediastinal lymph node, axillary lymph node, inguinal lymph node, spleen, kidney, adrenal gland, pancreas, cerebellum, and brainstem for NHP 1, and the lung, kidney, adrenal gland, pancreas, and submandibular lymph node for NHP 2. Endothelial cells, smooth muscle cells, dendritic cells, macrophages, fibroblast reticular cells and neurons of the cerebrum, cerebellum, and brainstem were predominantly stained.

**Table 3:** Immunohistochemistry.

	NHP 1	NHP 2	NHP 3	NHP 4
Tonsil (Follicles)	4,+,D	2,D	ND	ND
Tongue	2,+,M	2,M	ND	ND
Submandibular Salivary Gland	2,+,M	2,+,M	ND	ND
Nares	1,+,M	1,M	ND	ND
Larynx	2,+,M	1,+,M	ND	ND
Thyroid Gland	1,M	2,+,M	1,M	ND
Lung	4,+,D	4,+,D	1,M	ND
Heart	2,M	1,M	ND	ND
Thymus	4,+,M	Blush,M	ND	ND
Mediastinal Lymph Node	4,+,M	1,M	ND	ND
Aorta	1,F	Blush,D	ND	ND
Esophagus	1,+,M	1,+,M	ND	ND
Liver	1,+,M	2,+,M	ND	ND
Spleen (White Pulp)	4,+,D	2,D	ND	ND
Kidney	3,+,M	3,+,M	ND	ND
Urinary Bladder	2,M	1,+,M	ND	ND
Prostate Gland	1,F	1,F	ND	ND
Adrenal Gland	4,+,M	4,+,M	ND	ND
Mesenteric Lymph Node	1,+,M	2,+,M	ND	ND
Axillary Lymph Node	3,+,M	1,M	ND	ND
Inguinal Lymph Node	4,+,M	ND	ND	ND
Bone Marrow	1,M	1,M	ND	ND
Stomach	2,M	1,M	ND	1,M
Pancreas	4,M	4,+,M	ND	ND
Small Intestine	2,M	1,M	ND	1,M
Cecum	2,M	1,M	ND	1,M
Colon	2,M	1,M	ND	1,M
Haired Skin	1,M	ND	ND	ND
Skeletal Muscle	2,+,M	1,F	ND	ND
Cerebrum, Frontal Cortex	2,M	1,M	ND	4,M
Cerebrum, Corpus Striatum	1,M	1,M	ND	1,M
Cerebrum, Thalamus	1,M	1,M	ND	ND
Cerebrum, Mesencephalon	1,M	1,M	1,F	1,M
Cerebellum	4,M	1,M	1,F	ND
Meninges	1,M	1,M	ND	ND
Brainstem	4,F	1,M	ND	1,F
Tracheobronchial Lymph Node	1,M	2,M	ND	ND
Submandibular Lymph Node	ND	4,M	1,M	ND
Lip	ND	1,M	ND	ND

1 = 1–10 cells/high power field (hpf).

2 = 11–20 cells/hpf.

3 = 21–40 cells/hpf.

4 = >40 cells/hpf.

D = Diffuse.

F = Focal.

M = Multifocal.

+ = intense staining.

ND = Not detected.

Blush = staining was light and non-specific.

No prominent findings were appreciated by gross examination at necropsy for end of study survivors. The most prominent histopathologic lesions for these animals were mononuclear and lymphohistiocytic encephalitis and meningitis (affecting the cerebrum, cerebellum, and brainstem) (Fig. 11), systemic lymphohistiocytic vasculitis, and gliosis with rarefaction and spongiosis +/− necrosis; mild to minimal hepatic and renal lymphohistiocytic inflammation was noted for both animals. Additionally, NHP 4 had granulomatous inflammation in the lungs and sinus histiocytosis of the axillary, inguinal, and mesenteric lymph nodes. NHP 3 had fibrinoid necrosis of the liver, fibrinoid necrohemorrhagic pancreatitis, and mild lymphocytic inflammation of the lip, nares, and ciliary body of the eye. Syncytial cells were present in the spleen, tongue, stomach, submandibular salivary gland, urinary bladder, prostate gland, and thyroid gland for NHP 3. At PID 32, staining for viral antigen was absent from most tissues (Table 3); however, very strong staining (> 40 cells/ hpf) was observed in the frontal cortex of the cerebrum for NHP 4. Minimal staining (≤ 10 cells/hpf) was noted in the stomach, small intestine, cecum, colon, corpus striatum and mesencephalon of the cerebrum, and brainstem for NHP 4, and the thyroid gland, lung, mesencephalon of the cerebrum, cerebellum, and submandibular lymph node for NHP 3. The minimal, weak staining in many of the tissues in the end of study survivors could be specific but may also be background or nonspecific staining. Epithelial cells, macrophages, fibroblast reticular cells, and neurons of the cerebrum, cerebellum, and brainstem were predominantly stained.

## Discussion

Inoculation of African green monkeys with 2.5×10^4^ pfu of the Malaysia strain of NiV by the i.t. route did not result in uniform lethality; however, correlates of disease were present in all animals on this study. Our findings differ from a published report which suggested that a target dose of 2.0×10^4^ pfu of NiV-Malaysia by the i.t. route is uniformly lethal [22]. It should be noted that only one animal in that report actually received a dose close to the target dose of 2.0×10^4^ pfu [22]. In addition, a subsequent study using the same African green monkey model and virus strain to evaluate a vaccine against NiV included only one control animal that was infected with 1.0×10^5^ TCID_50_ (approximately 1.5×10^5^ pfu), and this animal was not viremic at the time that it was deemed moribund [24]. Both of these studies lacked the statistical power required to determine that a certain dose of NiV is uniformly lethal by the i.t. route in African green monkeys. Here, we performed a statistically powered study to evaluate the virulence of 2.5×10^4^ pfu of the Malaysia stain of NiV in African green monkeys infected by the i.t. route. Although only 50% of the animals on this study succumbed to disease, 75% developed clinical signs that included changes in responsiveness and respiratory difficulty, and 100% had evidence of NiV infection. Similar to NiV disease in humans [9–11,18,19], pneumonitis, systemic vasculitis, and coagulopathy were prominent findings in animals that succumbed, and significant histopathologic lesions were present in most tissues assessed, including lymphoid tissues (lymph nodes, spleen, and thymus) and the lungs. Virus antigen was often associated with areas of necrosis and/or inflammation in virtually all tissues assessed, confirming that the damage was directly related to NiV infection. Our results were also consistent with data presented for other models of NiV infection including ferrets, cats, and hamsters [20,25–27]. The data suggest that caution should be exercised when using the African Green model of NiV since 2.5×10^4^ pfu of the Malaysia strain of NiV is not uniformly lethal. Studies aimed at testing the effectiveness of vaccines or therapeutics must be appropriately powered to allow statistical relevance to be determined, with no less than 6 animals per group if survival is to be the primary criteria used to determine effectiveness.

Azotemia was present in animals that succumbed. Azotemia is often described as being either pre-renal, primary renal, or post-renal. Although we cannot definitively determine whether azotemia in this study was pre-renal or primary renal due to a lack of urine specific gravities, the presence of significant kidney lesions and prominent IHC staining of this tissue suggests a primary renal lesion. Serum glucose levels were also elevated for animals that succumbed. This could be related to pancreatitis observed for these animals, and is often seen in cases of shock. Only surviving animals developed eosinophilia by PID 12. We speculate that eosinophilia may represent a biomarker for a favorable prognosis; however, larger sample numbers will be required to confirm this hypothesis.

In addition to respiratory distress, neurologic disease is associated with NiV infection in humans, and relapse encephalitis occurs in approximately 7.5% of survivors and 3.4% of individuals who were initially asymptomatic [17,18,21]. Both animals that survived had histopathologic and immunohistochemical evidence of systemic NiV infection in the form of chronic vasculitis and prominent encephalitis and meningitis with concomitant immunohistochemical neuronal antigen staining. These findings are in agreement with findings associated with relapse encephalitis in human cases [18,21]. In the animals that succumbed, early histologic features of CNS pathology (primarily gliosis) were present, and viral antigen was found in the cerebrum, cerebellum, meninges, and brainstem; the lesions were often early on in their course and tended to be more subtle than neurologic lesions seen in animals that were euthanized at the end of the study.

In this study, viral RNA was detected in PBMCs and in throat, nasal, and rectal swabs of all animals. Viral RNA was also detected in plasma samples (data not shown), although plasma-associated NiV RNA was detected on fewer days and levels were 50–80 fold lower than levels in PBMCs. These data suggest that NiV transport in the blood is primarily cell-associated. Peak viral RNA titers in PBMCs were 50 fold higher for animals that succumbed compared to end of study survivors; significant differences in RNA titers in swabs were not observed. Additionally, protracted presence of viral RNA in blood (≥ 7 days) and swabs (≥ 11 days) was observed for survivors. The presence of infectious virus in throat, nasal, and rectal swabs was confirmed by plaque assay for animals that succumbed. Shedding of virus from the nose, throat, and rectum has been previously shown for the Malaysia strain of NiV in ferrets and Syrian hamsters, and virus has been found in respiratory secretions, throat swabs, and/or nasal swabs from human patients in Bangladesh and Malaysia [13–16,25,28]. Viral RNA was present in throat, nasal, and rectal swabs of African green monkeys described in the study by Geisbert *et al* [22], albeit to much lower levels. Our data support the published literature which demonstrate the potential for viral shedding by the oralpharyngeal or fecal routes.

**Fig 12:**
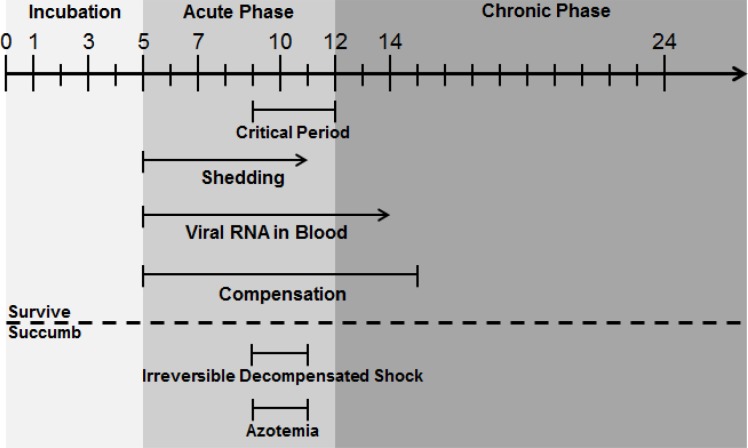
NiV progression in African green monkeys following i.t. exposure. A timeline of events during NiV infection in African green monkeys is shown. The timeline itself is in PIDs, with 0 being the day of virus exposure. Arrows were used when a certain condition was measured on the last blood collection day prior to end of study to suggest that the condition may exist beyond that study day. An arrow was also used at the end of the timeline to demonstrate that, although this study was terminated on PID 32, Chronic Phase could continue for an unknown length of time beyond that point. The broken horizontal line separates conditions seen in all animals, including those that survived, from conditions only seen in animals that succumbed.

We identified 3 phases of NiV disease in the African green monkey: Incubation (asymptomatic), Acute (symptomatic), and Chronic (Fig. 12). The Incubation Phase began after infection and lasted approximately 5 days; viral RNA in throat and nasal swabs was first detected during this phase (however, infectious virus detection was delayed to the beginning of Acute Phase and only detected in animals that succumbed), and some elevation in heart rate from baseline occurred. The Acute Phase began around PID 5 and lasted for about 7 days. This was the most dynamic phase of disease. Oropharyngeal shedding of live virus was detected as early as PID 5 and was concomitant with the presence of viral RNA in the blood. An initial fever response seen by PID 6–7 was followed by the appearance of viral RNA in rectal swabs. Only one animal in this study had measurable infectious virus in rectal swabs (NHP 1 on PID 9), but these data suggest that rectal shedding is a possibility during the latter part of the Acute Phase when viral load is highest. Increases in heart rate and respiratory rate seen in most animals during Acute Phase suggested responses to fever, to a developing hypoxic state, and/or to a state of compensatory shock. A critical period began around the 4^th^ day of Acute Phase (PID 9) and lasted until the end of Acute Phase (PID 12) where the most severe disease signs, such as hypotension, thrombocytopenia, erythropenia, and hypoalbuminemia were observed. These signs, seen in varying degrees in NHP 1–4, suggested coagulopathy and a transition to decompensated shock. Animals that succumbed appeared to enter a state of irreversible decompensated shock in which hypothermia, azotemia, hyperglycemia, and signs of liver disease developed. These animals rapidly progressed to a moribund state. For animals that survived, signs of shock, if present, began to resolve around PID 12, and the animals progressed to a Chronic Phase during which clinical signs of disease subsided and neutralizing antibody titers rapidly rose to high levels. Viral RNA was rarely detected in oropharyngeal swabs, and NiV RNA was still present in the blood through PID 14. By PID 15, most physiological parameters returned to baseline or near baseline levels. At some point during Chronic Phase, reactivation of infection in the brain occurred, and relapse encephalitis ensued. For the animals on this study, relapse encephalitis was not apparent clinically but was noted histopathologically. It is possible that these animals were at an early stage in the development of relapse encephalitis and, therefore, could have gone on to develop clinical signs if the study had continued beyond the pre-planned study end date.

The phases of NiV disease we describe for African green monkeys are similar to those observed in humans [9–11,17–19,21]. Based on the data described in this report and in the literature for human disease, it is evident that medical countermeasures against NiV should be evaluated for their ability to protect against both Acute (symptomatic) and Chronic (relapse) Phase disease. Further development of the African green monkey model is crucial as it provides a mechanism to address both the lethal as well as the chronic phase of the disease in a physiologically relevant model. However, until additional statistically powered dose studies are conducted to determine the most appropriate dose of NiV to use for medical countermeasure development, these types of studies should be appropriately balanced to account for the possibility of survival of control animals. The extensive data described herein can be used to better inform supportive care decisions during outbreaks. Understanding how physiological changes (such as changes in blood pressure or heart rate) relate to disease progression can help physicians decide on available treatment options that are most relevant and have the greatest likelihood of improving the patient’s condition, which could increase the comfort of the patient and possibly even improve their prognosis.

## Materials and Methods

### Animals

Animal research was conducted at the United States Army Medical Research Institute of Infectious Diseases (USAMRIID). Four adult, male, henipavirus-naïve *Chlorocebus aethiops* (African Green monkeys) of Caribbean origin were obtained from Worldwide Primates (Miami, FL). All animals had passed a semi-annual physical examination and were certified as healthy by a Veterinarian. Animals were acclimated in ABSL-4 animal rooms for 7 days prior to study initiation and housed in 4.4 square foot cages (27” L x 23.5” W x 34” H). Animals were individually housed during the in-life portion of the study due to safety concerns regarding social housing in biocontainment. During the in-life portion of the study, animals were provided 2050 Monkey Chow (Harlan Teklad, Frederick, MD), fruits, and water *ad libitum* via an automatic watering system and animals were given enrichment regularly as recommended by the Guide for the Care and Use of Laboratory Animals.

### Ethics statement

These experiments and procedures were reviewed and approved by the United States Army Medical Research Institute for Infectious Diseases Institutional Animal Care and Use Committee (IACUC). All research was conducted in compliance with the USDA Animal Welfare Act (PHS Policy) and other federal statutes and regulations relating to animals and experiments involving animals, and adheres to the principles stated in the Guide for the Care and Use of Laboratory Animals, National Research Council, 2011. The facility is fully accredited by the Association for Assessment and Accreditation of Laboratory Animal Care, International. The animals were provided food and water *ad libitum* and checked at least daily according to the protocol. All efforts were made to minimize painful procedures; the attending veterinarian was consulted regarding painful procedures, and animals were anesthetized prior to phlebotomy and virus infection. Following the development of clinical signs, animals were checked multiple times daily. When clinical observations and scores of animals reached defined levels based on the approved IACUC protocol, animals were euthanized under deep anesthesia to minimize pain and distress. Animals were humanely euthanized (when moribund or at the end of study) by intracardiac administration of a pentobarbital-based euthanasia solution under deep anesthesia in accordance with current American Veterinary Medical Association Guidelines on Euthanasia and institute standard operating procedures.

### Virus

The Malaysia strain of NiV was isolated from a patient from a 1998–1999 NiV outbreak in Malaysia [22,29], and was transferred to USAMRIID from the Centers for Disease Control and Prevention. The seed stock of virus used in this study was generated by passaging this material on Vero cells 3 times. On the day of challenge, PID 0, a stock of NiV was prepared by making the required dilutions to achieve a dose of 2.5×10^4^ pfu (target dose was 2.0×10^4^ pfu) in MEM Alpha GlutaMax-I (Life Technologies, Grand Island, NY) containing 10% heat-inactivated fetal calf serum (Life Technologies, Grand Island, NY).

### Virus Exposures

Virus exposures were performed by the i.t. route under anesthesia. A 5–12 French gauge feeding tube catheter was inserted into the trachea using a laryngoscope and the entire volume of virus (1 mL) was infused. Samples of the dilution series for preparation of the exposure agent were titrated by plaque assay to determine the actual dose delivered.

### Animal Observations and Euthanasia

Animals were evaluated cage side for signs of illness. Other observations such as biscuit/fruit consumption, condition of stool, and urine output were also documented, if possible. If more than 1 observation per day was necessary (after PID 0), each observation occurred ≥ 4 hr after the prior observation. Observations under anesthesia (physical examinations) occurred after cage side observations on PID-8, -1, 0, 1, 3, 5, 7, 10, 12, 14, and the day of disposition. Weights, blood collection, and collection of swab samples occurred during physical examinations.

Animals were humanely euthanized (when moribund or at the end of study) by intracardiac administration of a pentobarbital-based euthanasia solution under deep anesthesia in accordance with current American Veterinary Medical Association Guidelines on Euthanasia and institute standard operating procedures. Observations for euthanasia criteria assessment included responsiveness (0 = normal, 1 = mild unresponsiveness but active when approached, 2 = moderate unresponsiveness and withdraws when approached, 3 = severe unresponsiveness and does not withdraw when approached, 4 = unresponsive with no pain response), recumbency (0 = normal, 1 = occasional prostration, 2 = persistent prostration but normal when approached, 3 = persistent prostration), respiration (0 = normal, 1 = mildly labored, 2 = labored, 8 = agonal), bleeding (0 = none, 1 = mild, 2 = moderate, 3 = severe/copious), and seizures (0 = none, 1 = mild/petit mal, 2 = moderate/tonic-clonic, 3 = severe/tonic-clonic with or without delayed recovery, 4 = continuous). If the total euthanasia criteria score was ≥ 8 or if responsiveness = 4 or seizures = 4, the animal was considered moribund and was euthanized.

### Telemetry

Telemetry implants (T27F-1; Konigsberg Instruments, Inc., Pasadena, CA) were used to simultaneously monitor aortic blood pressure (ABP), left ventricular pressure (LVP), intrathoracic pressure (ITP), heart electrical activity (ECG), and core body temperature. African green monkeys with implants were placed into cages with ITS dipole antennae (Konigsberg Instruments, Inc., Pasadena, CA). These antennae were connected via coaxial cable to an eight-receiver TD15 base station (Konigsberg Instruments, Inc., Pasadena, CA). The base station was a general purpose signal processor that received the serially encoded analog signals collected from the implants and decoded them into parallel analog channels. The analog signals were then routed to an analog-to-digital converter, which converted the signals to raw digital data. These raw data files were converted into the Notocord NSS format using the CARecorder2NSS program (Notocord Inc., Newark, NJ). The data in the NSS files were processed and reduced using the Notocord-hem software platform (Notocord Inc., Newark, NJ). Reduced data in the NSS files was extracted into Microsoft Excel workbooks using Notocord-derived formula add-ins, and the 30 minute (min) averages were calculated for each parameter for each subject. Telemetry data collected in the six days prior to challenge was used as baseline, and provided the average and standard deviation (SD) for each 30 min daily time period of a 24 hour day.

### Necropsy

Necropsies were conducted by a veterinary pathologist on all animals in this study. The tissue samples were trimmed, routinely processed, and embedded in paraffin. Sections of the paraffin-embedded tissues 5 μm thick were cut for histology. For histology, slides were deparaffined, stained with hematoxylin and eosin (H&E), coverslipped, and labeled. For IHC, unstained sections were deparaffinized, rehydrated, subjected to a methanol-hydrogen peroxide block, rinsed, pre-treated with tris(hydroxymethyl)aminomethane/ Ethylenediaminetetraacetic acid buffer for 30 min at 95°C, blocked with 5% goat serum, and stained using a polyclonal rabbit anti-NiV antibody (USAMRIID #1294) followed by a horseradish peroxidase conjugated, anti-rabbit detection antibody. All sections were exposed to 3,3'-Diaminobenzidine substrate for 5 min, rinsed, counterstained with hematoxylin, dehydrated, and coverslipped.

### Clinical Pathology

For serum chemistries, whole blood was collected into Z Serum Clot Activator Greiner Vacuette tubes (Greiner Bio-One, Monroe, NC). Tubes were allowed to clot for 30–60 min and the serum separated in a centrifuge set at 1800 × g for 10 min at ambient temperature. The required volume of serum was removed for chemistry analysis using a General Chemistry 13 panel (Abaxis, Union City, CA) on a Piccolo Point-Of-Care Analyzer (Abaxis, Union City, CA). Serum was removed from the clot within 1 hour of centrifugation and was analyzed within 12 hours of collection.

For hematology, whole blood was collected into Greiner Vacuette blood tubes containing K3 EDTA as an anti-coagulant. Hematology was performed on the Hemavet 950 FS (Drew Scientific, Waterbury, CT) within 4 hours of collection. Following completion of the complete blood count analysis, 1.5 mL of whole blood was added to 1.5 mL of phosphate buffered saline (w/o calcium or magnesium) (PBS-/-; Life Technologies, Grand Island, NY) for PBMC isolation.

### PBMC Isolation

Three mL of whole blood/PBS−/− (1:1 mixture) was added to an Accuspin tube containing 3 mL of Histopaque-1077 as a cushion (Sigma-Aldrich, St. Louis, MO). The tubes were centrifuged at 800 × g for 20 min with no brake at ambient temperature. The PBMC band was removed into a 15 mL conical tube, washed with 13 mL of PBS-/-, and centrifuged at 200 × g for 7 min at ambient temperature. The liquid was removed from the pellet, and the pellet was suspended in 200 μL of PBS-/-; 600 μL of TRI Reagent-LS (Sigma Aldrich, St. Louis, MO) was added in preparation for RNA extraction and qRT-PCR.

### Preparation of Swabs for Virology

Swab samples were suspended in 1 mL of MEM 2.5 (Minimum Essential Medium [Life Technologies, Grand Island, NY] containing 2.5% heat-inactivated fetal calf serum [Life Technologies, Grand Island, NY], 1X L-glutamine [Life Technologies, Grand Island, NY), and 1X Penicillin/Streptomycin [Life Technologies, Grand Island, NY]) by vortex for 15 seconds followed by incubation at room temperature for 20 min. Clarification was performed by centrifugation at 14,000 × g for 30 seconds, and 200 μL of clarified supernatant was added to 600 μL of TRI Reagent-LS in preparation for RNA extraction and qRT-PCR. In addition, 200 μL of clarified supernatant was analyzed for infectious virus by plaque assay.

### qRT-PCR

Total RNA was extracted with TRI Reagent-LS, and the extracted nucleic acid was suspended in 30 μL of nuclease free water. cDNA was generated by using 10 μL of total nucleic acid extract with Superscript II and random hexamer priming (Life Technologies, Grand Island, NY). Real-time PCR was performed on 5 μL cDNA in TaqMan Universal PCR Master Mix (Life Technologies, Grand Island, NY) containing primers NiVN-Mal1051F—5′ AAT CGT GGT TAT CTT GAG CCT ATG T and NiVN-Mal1116R—5′ TGC CAT GTT CTG ATC AAT TCC T at 300 nM final concentration, and probe NiVN1079-Probe—5’ FAM TCA GAC TAG GCC AAA AAT CAG CAC GTC A-BHQ at 200 nM final concentration (Eurofins MWG Operon) (oligonucleotides were designed based on the NP gene sequence of GenBank Acc. no. AF212302). qRT-PCR was performed on a Step One Plus sequence detector (Life Technologies, Grand Island, NY) in a final volume of 25 μL, applying a standard cycling protocol of 50°C for 2 min, 95°C for 10 min, 45 cycles of 95°C for 15 sec, and 60°C for 1 min. Assays were run in duplicate and target copy numbers calculated based on Ct values in reference to serial dilutions of a calibrated plasmid standard containing the cloned target region.

### Plaque Assay

Challenge dose and infectious virus in swab samples were determined by Avicel plaque assay. Ten-fold dilutions of samples were made in MEM 2.5. Media on 6-well plates containing Vero cells (ATCC, Manassas, VA) at 80–90% confluency was decanted, and 200 μL of undiluted sample (swabs only) and each dilution was added (each sample was plated in triplicate). The cells were incubated at 37°C and 5% CO_2_ for 1 hr, with gentle rocking every 15 min. Avicel RC-591 (FMC BioPolymer, Philadelphia, PA) at a 2.5% concentration was mixed with an equal volume of 2X MEM (5% fetal bovine serum, 2X L-glutamine, and 2X penicillin/streptomycin), and 2 mL was added to each well. The cells were incubated for 4 days at 37°C and 5% CO_2_, stained with crystal violet (Sigma-Aldrich, St. Louis, MO), and plaques counted. The average number of plaques in triplicate wells was multiplied by 5 to determine pfu/mL for each dilution, and this value was multiplied by the dilution factor to determine pfu/mL of the starting sample.

### VZV-pseudotype Neutralization Assay

Multicycle VSV-pseudotype neutralization assays were performed as described previously [30], using a recombinant vesicular stomatitis virus (VSV) construct whose glycoprotein (G) gene was replaced by a red fluorescent protein (RFP) reporter gene and that was pseudotyped on NiV G and F glycoprotein expressing cells through cotransfection with the respective individual plasmids. Briefly, 293T cells were transfected with NiV G and F expression plasmids and a yellow fluorescent protein reporter plasmid. Four hours post transfection test sera and the NiV G and F pseudotyped virus were added. Wells were analyzed for RFP and YFP fluorescence after a 72 hour incubation at 37°C. Neutralization was determined by reduction in RFP signal (normalized for transfection efficiency indicated by YFP) compared to the control (cells and pseudotyped virus alone, no test serum added).
